# Revealing the multi-target destruction induced by proanthocyanidins against *Acetobacter* sp. at the molecular level

**DOI:** 10.3389/fmicb.2025.1624564

**Published:** 2025-07-04

**Authors:** Er-Fang Ren, Xiaoqin Feng, Yuanxin Feng, Kai Li, Xin-An Zeng, Qing-Hui Wen, Jin-Lin Cai, Zhong Han, Shan Chen, Debao Niu

**Affiliations:** ^1^College of Light Industry and Food Engineering, Guangxi University, Nanning, China; ^2^Guangxi Subtropical Crops Research Institute, Nanning, China; ^3^School of Food Science and Engineering, South China University of Technology, Guangzhou, China; ^4^School of Health, Jiangxi Normal University, Nanchang, China

**Keywords:** proanthocyanidins, cell membrane, membrane fatty acid, enzyme activity, genomic DNA, molecular docking

## Abstract

Proanthocyanidins, which are polyphenolic compounds resulting from the condensation of monomeric flavan-3-ols, exhibit antibacterial activity. This study aimed to investigate the impact of proanthocyanidins on the growth and cell membrane of *Acetobacter* sp., as well as explore their interaction with intracellular macromolecules to comprehensively elucidate the inhibitory mechanism against *Acetobacter* sp. The results revealed that proanthocyanidins effectively inhibited the growth of *Acetobacter* sp., with a minimum inhibitory concentration (MIC) of 2.5 mg/mL. Proanthocyanidins disrupted the cellular morphology and membrane integrity of *Acetobacter* sp., and affected the structure of cell membranes by interaction with membrane proteins. Meanwhile, exposure to proanthocyanidins led to an elevation in reactive oxygen species (ROS) levels within *Acetobacter* sp., causing oxidative damage to the cell membrane. Moreover, there was a modification in the composition of fatty acids within the cell membrane, characterized by an increased proportion of unsaturated fatty acids (UFAs), consequently enhancing membrane fluidity. In addition, proanthocyanidins caused a significant decrease in the activities of Alcohol dehydrogenase (ADH) and Aldehyde dehydrogenase (ALDH), and interacted with DNA through groove binding, thereby inhibiting cell function. In conclusion, this study provides evidence that proanthocyanidins can effectively inhibit the growth and reproduction of *Acetobacter* sp. by destroying cell membranes and affecting intracellular macromolecules.

## Graphical Abstract

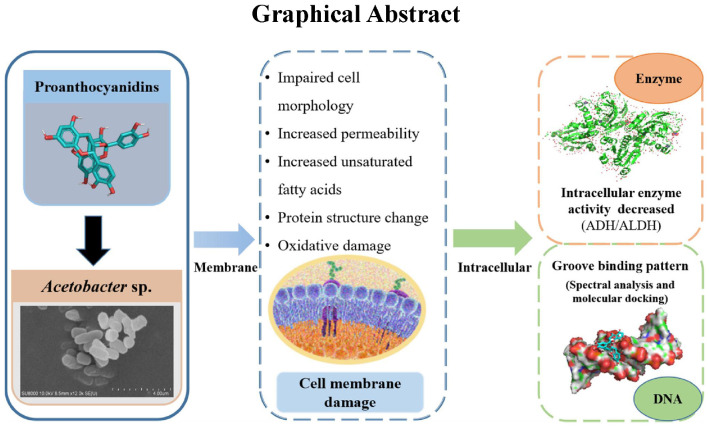

## 1 Introduction

*Acetobacter* sp., a typical Gram-negative brevibacterium, is widespread in orchard soils, grape surfaces and winemaking environments. This bacterium possesses the capability to transform ethanol produced by alcoholic fermentation into acetic acid, leading to an elevation of volatile acidity in wine (Valera et al., 2017; Niu et al., 2019a). Therefore, the control of *Acetobacter* sp. in wine throughout production has been the focus of the wine industry (Feng et al., 2022; Niu et al., 2021). The common control method to prevent wine spoilage is to add sulfur dioxide (SO_2_) with antibacterial properties (Lisanti et al., 2019). However, it is not enough to inhibit the growth of *Acetobacter* sp. due to strict limits on the amount of SO_2_ added, and *Acetobacter* sp. can still grow and multiply (Vavrinik et al., 2022). In addition, winemakers tend to reduce the use of SO_2_ to avoid negative effects on the physical health of consumers (Niu et al., 2020; Coulon and Seabrook, 2020). Hence, new preservative or microbial stabilization technologies need to be developed to address the urgent demand of the wine industry.

Previous studies have shown that a variety of natural functional substances such as Nisin, Chitosan and naringenin prevent microbial spoilage and can be used as substitutes for chemicals (Pei et al., 2016; Tedesco et al., 2022; Wang et al., 2017a). Proanthocyanidins are bioflavonoid compounds with a special molecular structure, which are polymerized by different amounts of catechin or epicatechin. Widely present in grape seeds and grape skins, they are a natural antioxidant with excellent antibacterial activity (Ulrey et al., 2014; Rauf et al., 2019). Exposure of *Listeria monocytogenes* to grape seed extract, which is abundant in proanthocyanidins, resulted in a notable reduction in the number of viable bacteria (Bisha et al., 2010). Moreover, it has been reported that proanthocyanidins derived from *Pelargonium sidoides* DC root extract could effectively suppress the growth of *Aggregatibacter actinomycetemcomitans, Staphylococcus epidermidis*, *Staphylococcus aureus* and *Escherichia coli*, and reduce their metabolic activity at the same time (Jekabsone et al., 2019). Therefore, proanthocyanidins showed promise as a natural preservative for use in wine production. Previous research has primarily focused on the specific antimicrobial effects of proanthocyanidins on *E. coli* and their disruptive impact on cell membranes. However, there is a paucity of research examining the impact of membrane components, including membrane proteins and fatty acids, on the cell membrane. Furthermore, research has demonstrated that the antibacterial mechanism of polyphenols is not a single model, in addition to destroying microbial cell membranes, it may also act on large molecules such as DNA, enzymes and proteins in cells (Wang et al., 2017b).

Therefore, in order to further reveal how proanthocyanidins affect the activity of microorganisms, *Acetobacter* sp., related to wine rancidity, was selected as the research object in this experiment, and the antibacterial properties and mechanisms of action of proanthocyanidins against *Acetobacter* sp. were discussed. In addition, the influence of proanthocyanidins on cell growth, cell morphology, membrane fatty acid composition and membrane protein, as well as intracellular macromolecules (enzymes and DNA) of *Acetobacter* sp. cells were investigated. This study aimed to explore the potential inhibitory mechanism of proanthocyanidins on *Acetobacter* sp., and to provide a theoretical basis for the utilization of proanthocyanidins in the context of wine microbial control.

## 2 Materials and methods

### 2.1 Microorganism and chealsmic

The following microorganism was employed in this research: one Gram-negative bacteria, *Acetobacter* sp.. It can be grown using acetic acid as a sole carbon source. We exploited this characteristic by isolating it from the rancidity of red wine and storing it at −80°C in the laboratory. Then, the culture was propagated in YG medium (1% glucose, 1% yeast extract powder, 2% ethanol, v/v) and incubated on a shaker (220 rpm, 30°C; HY-5, JinBo Equipment Industry Co., Jiangsu, China) for 24 h. The proanthocyanidins standard (purity, ≥ 95%, CAS:4852-22-6), extracted from grape seeds, was procured from Beijing Solarbio Science & Technology Co., Ltd. (Beijing, China).

### 2.2 Antibacterial effect of proanthocyanidins

#### 2.2.1 MIC determination

The determination of minimum inhibitory concentration (MIC) was slightly modified based on previous reports in the literature (Matuschek et al., 2018; Elshikh et al., 2016). *Acetobacter* sp. was cultured at 30°C in YG medium, from which an inoculum was obtained and adjusted to a bacterial density of approximately 10^7^ CFU/mL by using a 0.85% NaCl solution. The culture medium with different concentrations of proanthocyanidins was prepared by gradient dilution method. After inoculating the bacteria solution, the culture medium was incubated in a 96-well plate (30°C, 24 h). The optical density (OD_600_) value before and after culture was determined by a Universal microplate reader (Infinite E Plex). The difference between the two measurements was taken as the ordinate and the concentration of proanthocyanidins as the abscissa to draw a broken line graph. The MIC was defined as the lowest concentration of proanthocyanidins at which no bacterial growth was detected after 24 h of incubation. Then the sterile growth of bacterial suspension was cultured in agar plate medium for subculture. Following a 24-h incubation at 30°C, the minimum concentration of proanthocyanidins required to achieve 99.9% mortality of *Acetobacter* sp. was determined as the minimum bactericidal concentration (MBC).

#### 2.2.2 Determination of growth curve of *Acetobacter* sp.

The antibacterial effect of proanthocyanidins was evaluated by measuring the growth curve of *Acetobacter* sp. using ultraviolet spectrophotometry (Zhou et al., 2016; Liu et al., 2020). First, the *Acetobacter* sp. bacterial solution was cultured to the logarithmic phase (approximately 10^7^∼10^8^ CFU/mL) and then inoculating into YG medium at a 1% volume. Proanthocyanidins were introduced to the cultures, resulting in final concentrations of 1/8, 2/8, 3/8, and 4/8 of the MIC. Subsequently, the bacterial suspension was incubated on a shaker (30°C, 220 rpm). At specified intervals, appropriate samples were taken from the medium and the optical density at 600 nm was measured using a spectrophotometer over a period of 0–24 h. The growth rate (μ) of *Acetobacter* sp. grown in different concentrations of proanthocyanidins was calculated using the following equation:


$$\mu =\left(\ln I_{0}-\ln I_{\text{e}}\right)/\left(t_{0}-t_{\text{e}}\right)$$

where *I*_0_ and *I*_*e*_ are OD_600__*nm*_ values at culture time *t*_0_ and time *t*_*e*_, respectively.

### 2.3 Effect of proanthocyanidins on cell membrane

#### 2.3.1 Scanning electron microscope analysis

To investigate the morphological alterations in *Acetobacter* sp., SEM analysis was conducted by referring to previous methods (Han et al., 2020; Wang et al., 2021). The *Acetobacter* sp. were cultured in a liquid medium (YG) until they reached the logarithmic growth phase. The bacterial suspension concentration was adjusted to approximately 10^7^ CFU/mL, varying concentrations of proanthocyanidins were introduced, and the mixture was incubated (30°C, 4 h). Cells were collected centrifugally (6,000 rpm, 4°C, 5 min), washed three times with PBS (0.1 M, pH 7.4), and the precipitated thalli were stored overnight at 4°C with PBS buffers containing 2.5% (v/v) glutaraldehyde for cell fixation. The fixed cells underwent dehydration through sequential treatments with ethanol concentrations of 30, 50, 70, 90 and 100%, and the dehydrated bacteria were treated with tert-butanol twice. The sample was dripped onto the tin foil and dried using a freeze-dryer. Ultimately, the gold coating was applied to the samples through cathodic sputtering under vacuum conditions, and the morphological alterations were examined using SEM.

#### 2.3.2 Determination of cell membrane permeability

Based on the existing methods, the method of determining the release of nucleic acid and protein from *Acetobacter* sp. was modified slightly (Lv et al., 2011; Niu et al., 2019b). The *Acetobacter* sp. bacterial grew to the logarithmic phase in YG liquid medium. Cells were collected centrifugally (4,000 rpm, 4°, 5 min), followed by three washes with PBS (0.01 M, pH 7.4) before being resuspended. 10 mL bacterial suspension was taken, combined with different concentrations of proanthocyanidins, and incubated (30°C, 4 h). The suspension was centrifuged (4,000 rpm, 5 min) to separate the supernatant, which was then analyzed for optical density at 260 and 280 nm using an ultraviolet-visible spectrophotometer (China). Nucleic acids exhibited characteristic peaks at 260 nm, while proteins displayed 280 nm.

#### 2.3.3 Determination of oxidative damage to cell membrane

Dihydroethidium (DHE) is capable of entering cells without hindrance through the cell membranes of living cells, where it is oxidized by intracellular reactive oxygen species (ROS) to form ethidium oxide. Ethidium oxide can be incorporated into chromosome DNA, resulting in the production of red fluorescence (Zhu C. et al., 2014). The degree of red fluorescence observed can be used as an indicator of the amount and change in ROS content within the cells. The *Acetobacter* sp. were grown to the stationary phase, after which varying concentrations of proanthocyanidins were added and incubated at 30°C for 4 h. The suspended cells were then harvested by centrifugation (6,000 rpm, 4°C, 5 min), the supernatant was removed, and the cells were adjusted to 10^6^ CFU/mL cell suspension with PBS buffers. Then 1–2 μL dihydroethidium red fluorescent dye stock solution (5 mm) was introduced into the cell suspension (200 μL). The mixture was blown and mixed evenly and incubated in a dark environment at 37°C for 40 min. Following incubation, the solution was centrifuged to remove the staining solution, cleaned twice with PBS and suspended again, and the fluorescence intensity was determined using a fluorescence spectrophotometer. The excitation wavelength was set at 518 nm, while the emission wavelength was 610 nm. In addition, the quantification of superoxide dismutase (SOD) in *Acetobacter* sp. cells was performed using a commercially available kit from Beijing Solarbio Science & Technology Co., Ltd.

#### 2.3.4 Membrane proteins fluorescence spectrometry

Membrane proteins fluorescence experiments were slightly modified according to the reported methods (Ye et al., 2007). The *Acetobacter* sp. was cultured until it reached the late logarithmic phase. Subsequently, the culture medium was removed through centrifugation and washed with 0.85% NaCl solution for 3 times. The cells were then suspended in 0.85% NaCl solution, resulting in a bacterial solution with a density ranging from 10^9^ to 10^10^ CFU/mL. The bacterial solution was divided into several groups, with varying concentrations of potassium iodide (KI) or proanthocyanidins, and incubated (25°C, 2 h). The fluorescence emission spectra of the bacterial samples were then determined using fluorescence spectrometers with excitation wavelengths set at 258, 280, and 296 nm.

#### 2.3.5 Fatty acid composition of cell membrane

The *Acetobacter* sp. was inoculated with YG liquid medium containing varying concentrations of proanthocyanidins, and cultured until stationary phase (220 rpm, 30C°). The membrane fatty acids of *Acetobacter* sp. cells were extracted and methylated following the method described by Sasser (2006). Gas chromatogram-mass spectrometry (GC-MS) was employed to quantify and identify the fatty acids by comparing them with a standard mixture of methyl bacterate (Sigma-Supelco, Bellefonte, PA). The findings were presented as relative percentages of each fatty acid, calculated by determining the proportion of the subpeak area to the total peak area encompassing all peaks.

### 2.4 Effect of proanthocyanidins on enzyme activity

*Acetobacter* sp. was cultured in liquid medium (YG) to the logarithmic phase, then different concentrations of proanthocyanidins were added and incubated (30C°, 2 h). The cells were harvested through centrifugation (6,000 rpm, 4C°, 5 min) and washed twice with PBS (0.1 M, pH 7.4). The activities of ADH/ALDH in the bacterial suspension of *Acetobacter* sp., before and after proanthocyanidins treatment were determined using a corresponding activity detection kit (Beijing Solarbio Science & Technology Co., Ltd.). The enzymatic activity was expressed as the relative residual activity (RRA, %):


$$RRA\left(\%\right)=A_{\text{e}}/A_{0}\times 100\%$$

where *A*_0_ and *A*_*e*_ represent the enzyme activity before and after proanthocyanidins treatment respectively.

### 2.5 Detection of proanthocyanidins binding to DNA

The genomic DNA of *Acetobacter* sp. was extracted by the previously reported method. Following extraction, the concentration and purity of the DNA were assessed utilizing a Universal microplate reader (Infinite E Plex). DNA concentration was quantified by absorbance at 260 nm. The purity of the DNA sample was assessed based on the ratios of OD_260nm_/OD_280nm_ and OD_260nm_/OD_230nm_ (1.8 ≤ OD_260nm_/OD_280nm_ ≤ 2.0, 2.0 ≤ OD_260nm_/OD_230nm_ ≤ 2.2). The competitive binding experiment was modified slightly based on the previously reported methodology (Ebrahimipour et al., 2015; Rehman et al., 2015). In 0.01 M PBS (pH 7.4), proanthocyanidins were sequentially introduced to the complexes of EB-DNA, with fixed concentrations of DNA (45 μg/mL) and EB (1.25 μg/mL). Following a 30-min incubation period at 25°, emission spectra within the range of 550–750 nm were recorded when excited at 530 nm.

### 2.6 Molecular docking

The crystal structure of DNA (PDB ID: 453D) was obtained from the Protein Data Bank at the Research Collaboratory for Structural Bioinformatics (RCSB). The PDB file required for docking proanthocyanidins with DNA molecules was generated by coupling with Auto Dock-Vina and ChemBio 3D software. The ChemBio 3D program was first applied to optimize the 3D structure of proanthocyanidins, which was then read by Auto Dock-vina, and the preparation of proanthocyanidins and DNA molecules before docking was performed in Auto Dock-vina. Molecular docking analysis was conducted using the Auto Dock-Vina program, which incorporates the Lamarckian Genetic Algorithm (LGA) for its calculations (Mukherjee and Singh, 2017). The grid box size was chosen to be large enough to contain the complete DNA dodecamer d(CGCGAATTCGCG)_2_. In the docking simulation, the conformation exhibiting the lowest binding energy was selected from a set of 10 different conformations. This corresponding minimum energy structure was then used for subsequent analysis. The PyMOL software package was used to obtain a visual 3D model of the docking results.

### 2.7 Statistical analysis

The experiments were conducted in triplicate. SPSS 19.0 software was used for one-way analysis of variance (ANOVA) in order to assess the presence of significant variations among the treatments (*p* < 0.05). Data were documented in the form of mean ± standard deviation. The graphics are created by Origin software.

## 3 Results and discussion

### 3.1 Inhibition of proanthocyanidins on *Acetobacter* sp. growth

**FIGURE 1:**
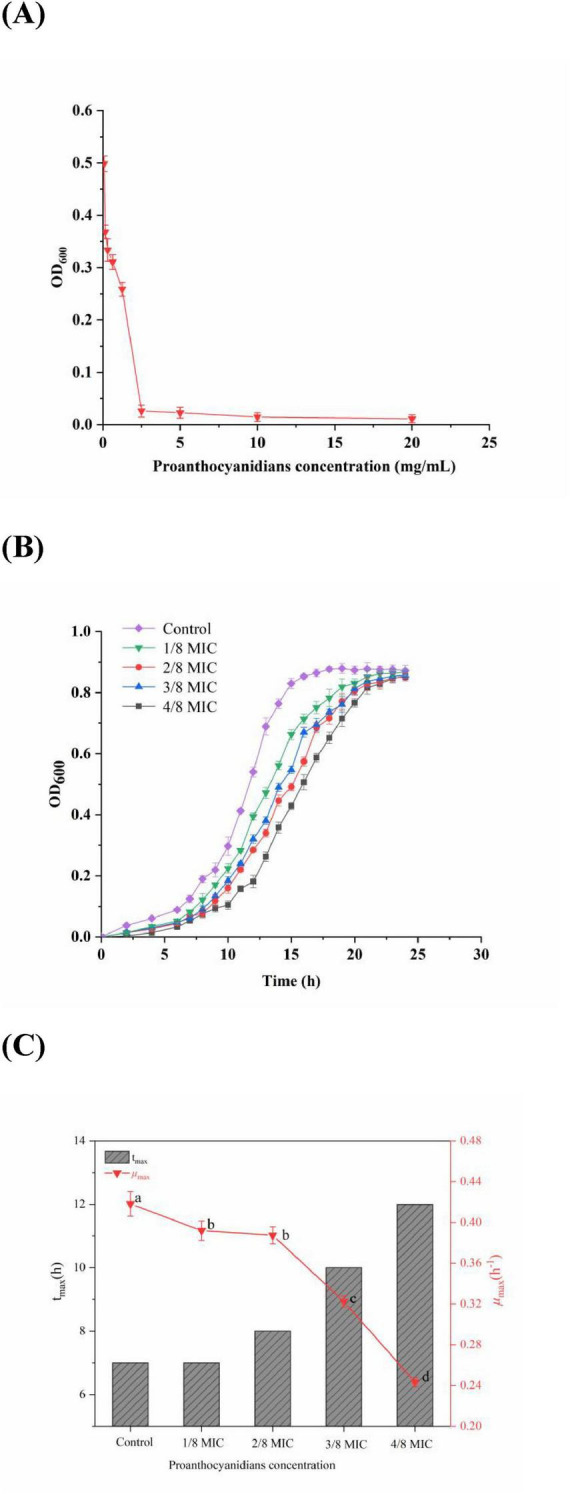
**(A)** Antibacterial activity; **(B)** growth curves; **(C)** maximum growth rate (μ_max_) and the time to reach the μ_max_ of *Acetobacter* sp. cultivated in the medium with the concentration of proanthocyanidins at 0 (Control), 1/8 MIC, 2/8 MIC, 3/8 MIC and 4/8 MIC. Different letters indicate significant differences (*p* < 0.05).

The MIC is considered as a standard to measure the antibacterial properties of natural substances (Matuschek et al., 2018). Figure 1A illustrated the inhibitory effects of proanthocyanidins on the growth of *Acetobacter* sp. as a function of varying concentrations. The findings revealed that proanthocyanidins demonstrated notable antibacterial efficacy against *Acetobacter* sp.. When *Acetobacter* sp. were cultured in an environment containing proanthocyanidins at concentrations of 2.5 mg/mL or higher for 24 h, the OD_600_ value of *Acetobacter* sp. was essentially unchanged, indicating significant growth inhibition of *Acetobacter* sp. at this concentration. On the contrary, when the concentration of proanthocyanidins was lower than 2.5 mg/mL, the OD_600_ value increased sharply. Therefore, the MIC of proanthocyanidins against *Acetobacter* sp. could be determined to be 2.5 mg/mL. In addition, the MBC of proanthocyanidins was 20 mg/mL, indicating that proanthocyanidins had strong bactericidal activity against *Acetobacter* sp.. In contrast, the MIC values of grape seed proanthocyanidins extract against *Actinomyces viscous* and *Streptococcus mutans* were 8.0 and 12.5 mg/mL, respectively, which were slightly higher than that in this experiment (Liu et al., 2011; Wang and Wang, 2017).

To provide additional validation of the efficacy of proanthocyanidins against *Acetobacter* sp., a growth curve analysis was performed for *Acetobacter* sp.. According to Figure 1B, *Acetobacter* sp. in the control group grew slowly from 0 to 7 h, followed by an acceleration from 7 to 15 h. Then the growth rate slowed down and reached its peak optical density value of 0.878 at 20 h. In addition, after exposure to proanthocyanidins, a significant delay in the adjustment period of *Acetobacter* sp. was observed, which was similar to the impact of cinnamaldehyde on the growth curve of drug-resistant *Aeromonas hydrophila* (Yin et al., 2020). Moreover, within a certain range, the higher the concentration of proanthocyanidins, the longer it took for *Acetobacter* sp. to enter the logarithmic phase and reach the stationary phase, but the OD_600_ values of the stationary phase showed little difference. According to Figure 1C, the μ_*max*_ of *Acetobacter* sp. exhibited varying degrees of decrease with increasing proanthocyanidins concentration. The μ_*max*_ of *Acetobacter* sp. decreased to 0.243 at 4/8 MIC and the time required to reach μ_*max*_ was delayed to 12 h, indicating the potent antibacterial activity of proanthocyanidins against *Acetobacter* sp., thus resulting in a significant retardation in its growth cycle.

### 3.2 Morphological changes of *Acetobacter* sp. cells

**FIGURE 2:**
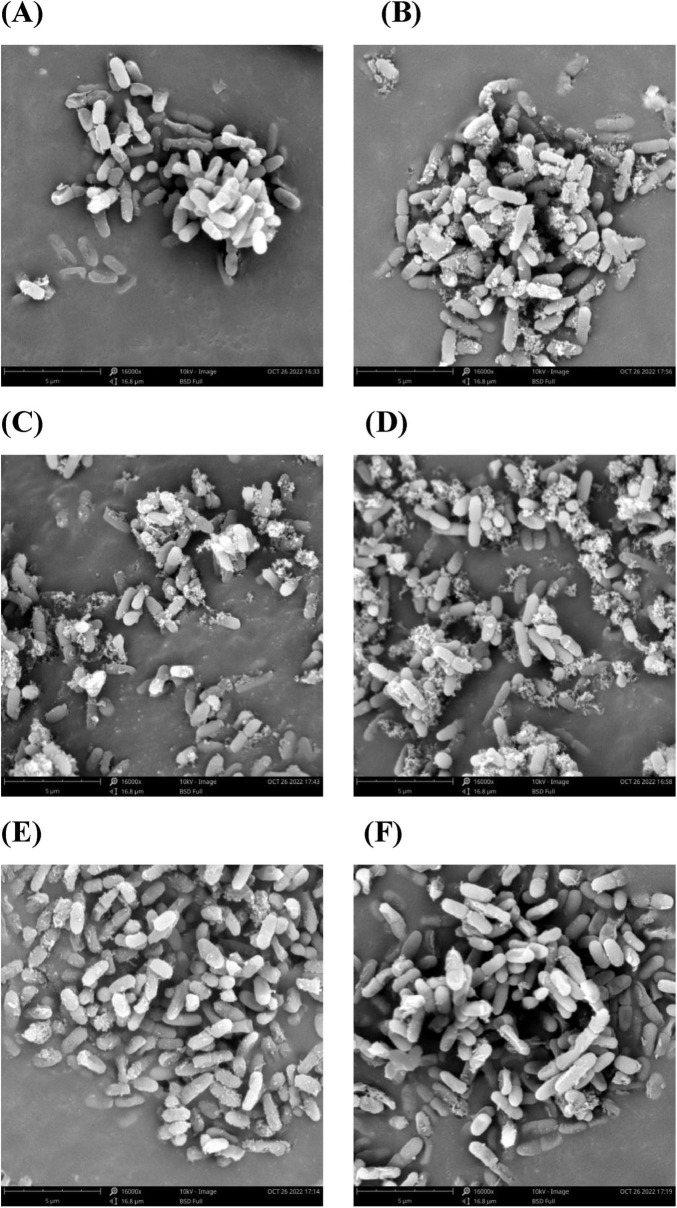
Morphology of *Acetobacter* sp. cells as examined under a scanning electron microscope (SEM). *Acetobacter* sp. treated with proanthocyanidins at 0 **(A)**, Control 1/8 MIC **(B)**, 1/4 MIC **(C)**, 1/2 MIC **(D)**, 1 MIC **(E)** and 2 MIC **(F)**.

The cellular membrane is the boundary between the internal and external environment of the cell. When cells are subjected to strong external stimuli such as antibiotics, cell morphology often changes accordingly. Therefore, SEM was used to further elucidate the effect of varying concentrations of proanthocyanidins on *Acetobacter* sp. and the results were depicted in Figure 2. The untreated bacterial cells exhibited a smooth and rod-shaped morphology with intact structural integrity. They displayed uniformity in both size and distribution, devoid of apparent deformations (Figure 2A). In contrast, *Acetobacter* sp. treated with proanthocyanidins had obvious morphological damage. Compared with the smooth cell surface of the untreated group, binding of proanthocyanidins to the cell surface of *Acetobacter* sp. was observed when cultured with 1/8, 1/4, and 1/2 MIC proanthocyanidins for 4 h. This implied that proanthocyanidins may impede the normal functioning of *Acetobacter* sp.’s cell membrane, thereby affecting its growth. Meanwhile, a fraction of the cells exhibited perforations, while others displayed shortened and fractured morphology with increased cell stacking and adhesion (Figures 2B–D). Following treatment with proanthocyanidins at 1 and 2 MIC, the *Acetobacter* sp. cells underwent significant alterations in morphology, displaying pronounced damage. Some bacterial cells exhibited deformation, shrinkage, and malformation (Figures 2E,F). The morphological changes of the *Acetobacter* sp. may be attributed to the impact of proanthocyanidins on membrane integrity and permeability, resulting in the release of intracellular materials such as ions, proteins and genetic materials, ultimately leading to bacterial cell wall lysis, membrane instability, and abnormal morphology. The SEM results revealed that proanthocyanidins induced alterations in the cellular morphology, disrupted the normal functioning of the cell membrane, and thus exerted inhibitory effects on the growth of *Acetobacter* sp.

### 3.3 Alterations in membrane permeability of *Acetobacter* sp.

**FIGURE 3:**
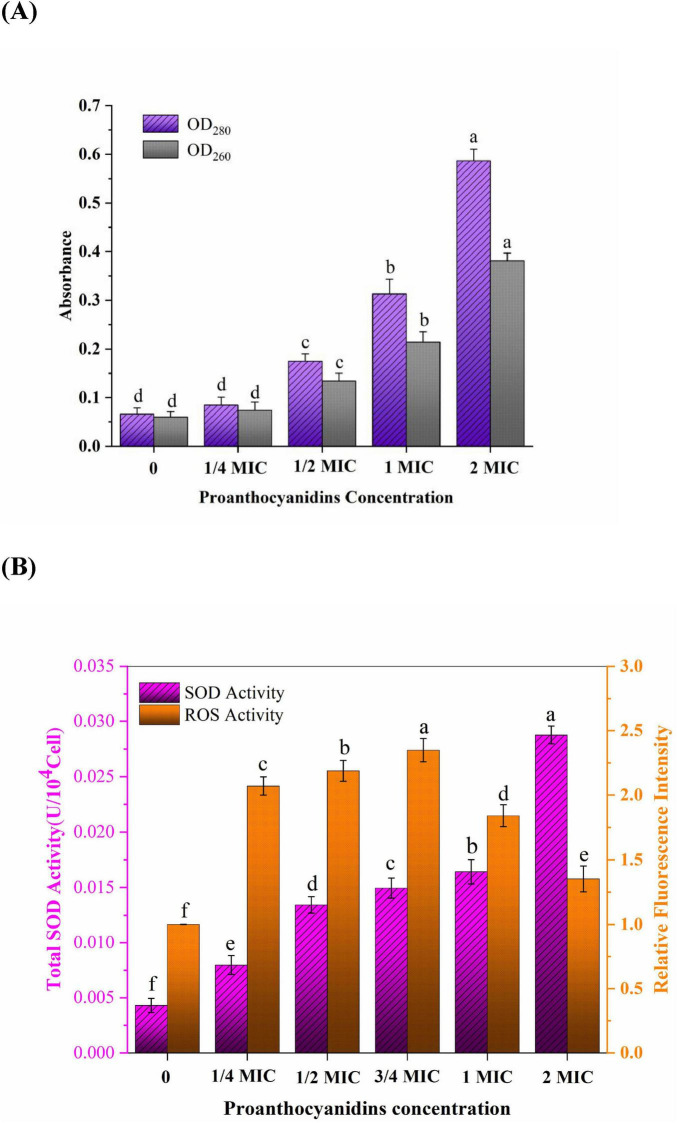
**(A)** Intracellular nucleic acids (OD_260_) and proteins (OD_280_) leakage of *Acetobacter* sp. treated with different proanthocyanidins concentrations. **(B)** Changes of ROS and SOD activity in *Acetobacter* sp. cultured with different concentrations of proanthocyanidins. Different letters indicate significant differences (*p* < 0.05).

To assess the impact of proanthocyanidins on the permeability of *Acetobacter* sp. membranes, intracellular proteins and nucleic acids leakage were measured. The release of nucleic acids and proteins into the supernatant of *Acetobacter* sp. after proanthocyanidins treatment for 4 h was shown in Figure 3A. Nucleic acids exhibited characteristic peaks at 260 nm, while proteins displayed peaks at 280 nm, with their concentrations being directly proportional to the absorption values. The OD_260_ values for *Acetobacter* sp. treated with 1/2 and 2 MIC were 2.23 and 6.35-fold higher, respectively, compared to the control group, while the OD_280_ values exhibited a respective increase of 2.42 and 8.28-fold. Figure 3A indicated that as the concentration of proanthocyanidins increases, the nucleic acids and proteins leaked by *Acetobacter* sp. also increased continuously. Similarly, the light density of *V. cholerae* suspensions treated with flavonoids glycosides increased significantly at 260 nm, indicating a loss of nucleic acids through the damaged cell membrane (Tagousop et al., 2018). The measurement of cell leakage markers, specifically absorbance at 260 and 280 nm, has been demonstrated as an effective indicator for assessing membrane permeability. This approach reflects the integrity of cell membranes and the release of internal components (Bajpai et al., 2013; Moghimi et al., 2016). Cell membrane permeability and integrity are crucial factors that influence the normal growth and metabolic processes of bacteria. The results of this study indicated that proanthocyanidins damaged cell membranes, affected their permeability and integrity, resulted in the leakage of nucleic acids, proteins and other intracellular macromolecules, and seriously led to cell death.

### 3.4 Oxidative stress of proanthocyanidins to *Acetobacter* sp. membrane

Oxidative stress refers to the excessive generation of intracellular ROS, leading to detrimental effects on lipids, DNA, and proteins and thus destroying the oxidation and reduction balance in the cell. Antibiotics not only play an antibacterial role by binding to specific targets, but also cause oxidative stress to bacteria by inducing the accumulation of ROS (Wu et al., 2022; Xu et al., 2024). Therefore, the oxidative stress of proanthocyanidins on *Acetobacter* sp. cell membrane was further investigated (Figure 3B). ROS play a role in diverse cellular processes encompassing cell growth, proliferation, development and differentiation, senescence, apoptosis, as well as various physiological and pathological phenomena (Wu et al., 2022). It was not difficult to find that the relative fluorescence intensity representing ROS content gradually increases with escalating concentrations of proanthocyanidins. The findings suggested a greatly stimulation of ROS generation in *Acetobacter* sp. by proanthocyanidins. At a concentration of 3/4 MIC, the relative fluorescence intensity was observed to be 2.35-fold higher than that of the control group. The substantial accumulation of ROS may be one of the reasons why cells gradually lose the ability to function normally. Interestingly, the relative fluorescence intensity of ROS increased first and then decreased. Upon surpassing a concentration of 1 MIC, the relative fluorescence intensity displayed a declining trend, it remained higher than that observed in the control group. This phenomenon can be attributed to the high concentration of proanthocyanidins, which induces a rapid decline in the activity or even mortality of certain *Acetobacter* sp.. The DHE fluorescence probe used for detection was only loaded into living cells, resulting in a reduction in the detected fluorescence intensity.

SOD is a vital antioxidant enzyme in living organisms, serving as a crucial scavenger of oxygen-free radicals. It plays an important role in catalyzing the disproportionation reaction of superoxide anion (O_2_^–^) and maintaining internal environment homeostasis (Rapacka-Zdonczyk et al., 2021). The activity of SOD in *Acetobacter* sp. is frequently employed for assessing the resistance of microorganisms to oxidative damage induced by adverse external conditions. With increasing concentrations of proanthocyanidins during incubation, the activity of SOD in *Acetobacter* sp. a noticeable upward trend was observed in the SOD activity, and the total SOD activity even reached 0.02876 U/10^4^Cell at 2 MIC, which was 6.7 times that of the untreated group. The findings demonstrated that *Acetobacter* sp. produced a large amount of O_2_^–^ under the action of proanthocyanidins. These O_2_^–^ attacked the polyunsaturated fatty acids in the cell membrane and caused lipid peroxidation, which induced the cells to resist by increasing the activity of total SOD (Johnson and Hug, 2019).

### 3.5 Effect of proanthocyanidins on membrane proteins

**FIGURE 4:**
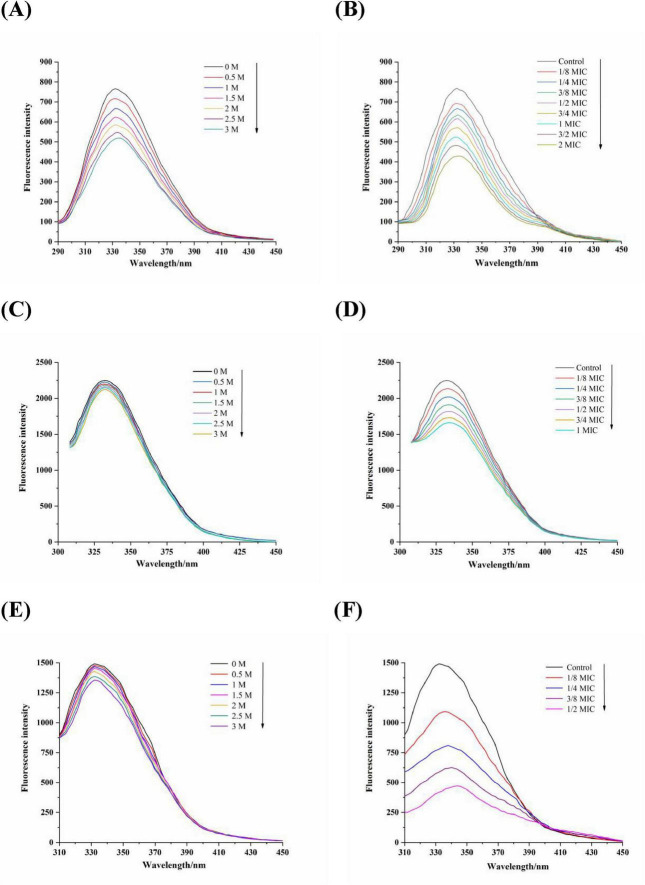
Fluorescence spectra of amino acids residues Phe **(A,B)**, λ_ex_ = 258 nm), Trp **(C,D)**, λ_ex_ = 280 nm), Tyr **(E,F)**, λ_ex_ = 296 nm) of *Acetobacter* sp. cells membrane proteins in the various concentrations of KI (**A**, **C** and **E**) and proanthocyanidins (**B**, **D** and **F**), respectively.

There are a large number of membrane proteins composed of amino acids on the bacterial cell membrane, in which tryptophan (Trp), tyrosine (Tyr), and phenylalanine (Phe) are the main fluorescence groups of membrane proteins (Ye et al., 2007). The positioning of these residues within the proteins can be determined by employing KI, a fluorescence quencher that effectively suppresses the fluorescence emitted by surface residues present in membrane proteins, while leaving the fluorescence spectra of internal residues unaffected. Figures 4A,C,E illustrated the impact of KI on the fluorescence emission spectra of Phe, Trp and Tyr residues with the fixed excitation wavelengths of 258, 280, and 296 nm, respectively. As the concentration of KI increased, a significant decrease in fluorescence intensity was observed for Phe while no apparent quenching effect was observed for Trp and Tyr residues. This revealed that Phe residues are primarily found on the exterior of the membrane proteins in *Acetobacter* sp., whereas Trp and Tyr residues are predominantly situated within the membrane (Ye et al., 2007). Figures 4B,D,F showed the fluorescence spectra of Phe, Trp, and Tyr residues when exposed to varying concentrations of proanthocyanidins. It was observed that the maximum emission intensity for these residues significantly diminished as the concentration of proanthocyanidins increased, accompanied by a red-shift phenomenon. This indicated that proanthocyanidins interact with membrane proteins of *Acetobacter* sp., leading to conformational changes of membrane proteins and causing the membrane proteins to unfold and expose the internal hydrophobic residues. As a result, more internal Phe, Trp and Tyr residues were exposed to the membrane proteins surface, facilitating increased interaction between proanthocyanidins and these residues for fluorescence quenching. The results implied that proanthocyanidins have the potential to influence the cellular membrane structure by interacting with proteins on *Acetobacter* sp.’s cell membrane.

### 3.6 Evolution of membrane fatty acids in *Acetobacter* sp.

The antibacterial effect of antibacterial substances is attributed to their ability to disrupt and impair the structure and fluidity of the cell membrane, as evidenced by numerous studies (Zhu B. et al., 2014). The alteration in membrane fluidity primarily resulted from variations in the fatty acid composition of the cell membrane. The membrane fatty acid detection results of *Acetobacter* sp. were presented in Table 1, in which a total of 13 fatty acids were identified. The predominant fatty acid components in *Acetobacter* sp. cell membrane were tetradecanoic acid (C14:0), 2-hydroxy-tetradecanoic acid (2-OH C14:0), Hexadecanoic acid (C16:0), 2-hydroxy Palmitic Acid (2-OH C16:0), 3-hydroxy Palmitic Acid (3-OH C16:0), stearic acid (C18:0), 9-Octadecenoic Acid (C18:1ω9), cis-9,10-Methyleneoctadecanoic Acid (C19:Cyclo). It should be noted that more than 55% of the fatty acids in the untreated *Acetobacter* sp. were unsaturated fatty acids (UFAs), which increased with increasing concentrations of proanthocyanidins. When cultured with 3/4 MIC proanthocyanidins, the maximum proportion of UFAs in the membrane of *Acetobacter* sp. reached 62.84%. It has been shown that UFAs increase in response to adverse environments such as low growth temperatures, acid intimidation, ethanol intimidation and oxidative stress (Alvarez-Ordonez et al., 2008). For example, the UFAs content of *E. coli* and *Salmonella Typhimurium* significantly increased when grown in a medium containing hexanal and carvacrol (Luz et al., 2014; Patrignani et al., 2008). Meanwhile, the proportion of saturated fatty acids (SFAs) in cell membranes decreased from 29.64 to 21.23% when cultured with 3/4 MIC proanthocyanidins. Generally, the increase in the content of UFAs tends to increase the membrane fluidity, whereas SFAs and cyclic fatty acids contribute to a more organized and compact membrane structure, resulting in decreased membrane fluidity (Di Pasqua et al., 2006). This implied that proanthocyanidins influence the fatty acid composition of *Acetobacter* sp. cell membranes, potentially leading to increased membrane fluidity.

**TABLE 1:** Cell membrane fatty acids composition of *Acetobacter* sp. grown in the medium with different concentrations of proanthocyanidins.

Fatty acids	Total composition (%) at different proanthocyanidins concentrations			
	0	1/4 MIC	1/2 MIC	3/4 MIC
C12:0	0.22 ± 0.02^bc^	0.29 ± 0.02^a^	0.17 ± 0.04^c^	0.24 ± 0.03^ab^
C14:0	2.25 ± 0.11^c^	3.40 ± 0.08^b^	3.73 ± 0.11^a^	3.67 ± 0.13^a^
C14:0 (2-OH)	4.17 ± 0.09^d^	4.61 ± 0.12^c^	5.58 ± 0.09^b^	5.86 ± 0.16^a^
C15:0	0.14 ± 0.04^b^	0.34 ± 0.06^a^	0.13 ± 0.06^b^	0.17 ± 0.03^b^
C16:0	15.29 ± 0.07^a^	14.28 ± 0.04^b^	13.72 ± 0.10^c^	11.87 ± 0.14^d^
C16:0 (2-OH)	5.68 ± 0.24^c^	5.89 ± 0.27^c^	6.53 ± 0.28^b^	7.11 ± 0.34^a^
C16:0 (3-OH)	2.36 ± 0.12^a^	2.43 ± 0.05^a^	2.05 ± 0.07^b^	1.71 ± 0.08^c^
C16:1ω9	0.71 ± 0.15^a^	0.60 ± 0.08^a^	0.38 ± 0.03^b^	0.23 ± 0.06^b^
C17:0	0.15 ± 0.08^b^	0.46 ± 0.04^a^	0.15 ± 0.02^b^	0.22 ± 0.10^b^
C18:0	11.58 ± 0.22^a^	8.02 ± 0.17^b^	7.29 ± 0.16^c^	5.06 ± 0.21^d^
C18:1ω9	56.19 ± 0.31^d^	57.91 ± 0.46^c^	58.91 ± 0.53^b^	62.61 ± 0.59^a^
C18:0 (3-OH)	0.77 ± 0.05^c^	1.32 ± 0.05^a^	0.95 ± 0.06^b^	0.94 ± 0.03^b^
C19:cyclo	0.48 ± 0.04^a^	0.45 ± 0.03^a^	0.40 ± 0.10^ab^	0.30 ± 0.07^b^
UFAs	56.90 ± 0.46^c^	58.52 ± 0.54^b^	59.29 ± 0.56^b^	62.84 ± 0.65^a^
SFAs	29.64 ± 0.54^a^	26.79 ± 0.41^b^	25.19 ± 0.49^c^	21.23 ± 0.64^d^
HFAs	12.98 ± 0.50^c^	14.25 ± 0.44^b^	15.12 ± 0.50^ab^	15.63 ± 0.61^a^
CFAs	0.48 ± 0.04^a^	0.45 ± 0.03^a^	0.40 ± 0.10^ab^	0.30 ± 0.07^b^

Different letters in the same line indicate significant differences (*p* < 0.05). HFAs and CFAs represent hydroxy fatty acids and cyclic fatty acids, respectively.

### 3.7 Changes in intracellular enzyme activity of *Acetobacter* sp.

**FIGURE 5:**
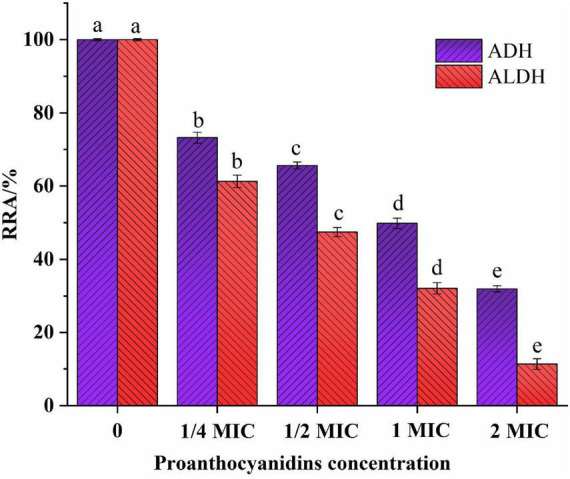
Effect of proanthocyanidins on relative residual activity (RRA) of intracellular enzymes (ADH and ALDH) of *Acetobacter* sp.. Different letters indicate significant differences (*p* < 0.05).

Some studies have shown that the antibacterial mechanism of polyphenols is not a single model, in addition to destroying microbial cell membranes, it may also act on biomacromolecules such as enzymes and DNA in cells (Wang et al., 2017b). ADH/ALDH, as crucial acidogenic enzymes in acetic acid bacteria, is able to convert ethanol into acetic acid, leading to an increase in volatile acidity in wine. The relative residual activities (RRA) of major intracellular enzymes (ADH/ALDH) of *Acetobacter* sp. were shown in Figure 5. The RRA of ADH/ALDH in *Acetobacter* sp. exhibited a declining trend as the concentration of proanthocyanidins treatment increased. The RRA of ADH and ALDH was 73.24 and 61.25% respectively when 1/4 MIC proanthocyanidins were added for 2 h. Gradually, With the increase of proanthocyanidins concentration to 2 MIC, the activities of two major enzymes decreased to 31.93 and 11.35%, respectively. These results demonstrated that proanthocyanidins intimidation significantly impacted the RRA of enzymes (ADH/ALDH) in *Acetobacter* sp.. Higher concentrations of proanthocyanidins corresponded to lower RRA values for these intracellular enzymes. In addition, ALDH was more sensitive to proanthocyanidins at the same concentration, and the reduction of enzyme activity was more severe (Figure 5). It was confirmed that proanthocyanidins not only impacted the cellular membrane structure and function of *Acetobacter* sp., but also influenced intracellular enzyme activity, and had an inhibitory effect on the acid-producing capacity of *Acetobacter* sp.

### 3.8 Binding of proanthocyanidins to DNA of *Acetobacter* sp.

**FIGURE 6:**
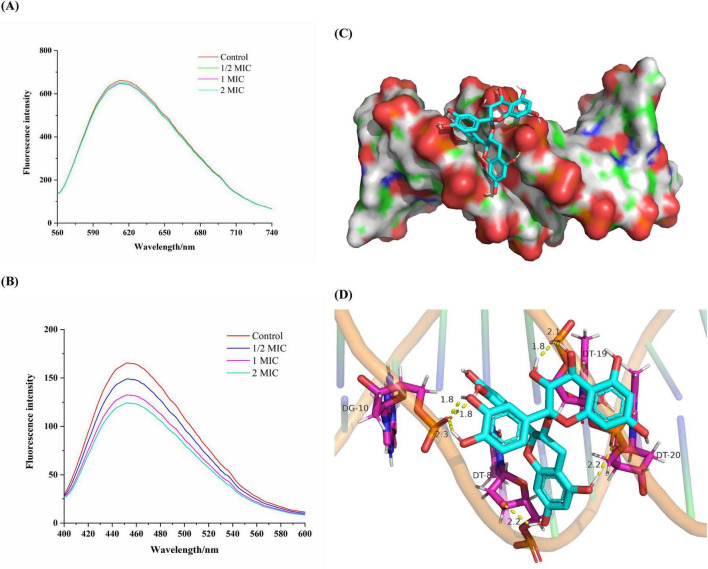
Fluorescence spectra of the EB-DNA **(A)** and Hoechst-DNA **(B)** system with increasing concentrations of proanthocyanidins. **(C)** Molecular docked structures of proanthocyanidins complexed with DNA. The figure represented minor groove binding of proanthocyanidins with dodecamer d(CGCGAATTCGCG)_2_ (PDB ID: 453D). **(D)** The possibility of hydrogen bonds formed by proanthocyanidins binding to DNA.

Competitive binding experiments were conducted using ethidium bromide (EB) and Hoechst-33258 to further elucidate the interaction pattern between proanthocyanidins and the DNA of *Acetobacter* sp.. EB is a cationic conjugated planar molecule with weak fluorescence intensity in aqueous environments, but can greatly enhance the fluorescence intensity of DNA due to embedding in DNA (Hu et al., 2019). As a result, EB has been employed as an effective DNA fluorescent probe to elucidate the interaction between DNA and small molecules or proteins. As shown in Figure 6A, the incorporation of proanthocyanidins into the EB-DNA system exhibited negligible impact on the fluorescence intensity of the EB-DNA complex, which indicated that proanthocyanidins were incapable of displacing EB from its association with DNA (Li et al., 2020). Hence, it was proved that the binding mode between proanthocyanidins and DNA is non-intercalation (Liu et al., 2015).

In another experiment, Hoechst-33258, as a fluorescent probe, was observed to bind specifically to DNA through groove mode, resulting in enhanced fluorescence characteristics upon binding (Guan et al., 2006). When molecules with a groove binding pattern compete with Hoechst for binding sites on DNA, the fluorescence intensity will be greatly reduced (Rehman et al., 2015). A significant fluorescence quenching was observed when proanthocyanidins were added into the Hoechst-DNA system (Figure 6B). As the concentration of proanthocyanidins increased, a significant reduction in fluorescence intensity was noted compared to the control group. This observation suggested that both proanthocyanidins and Hhoechst-33258 are capable of binding to DNA and competing for the binding sites, which ultimately led to a decrease in the fluorescence intensity of the Hoechst-DNA complex. Thus, the experiments demonstrated that proanthocyanidins exhibit groove binding mode interaction with DNA and can bind to the Hoechst-DNA complex, effectively competing with Hoechst for DNA binding sites in *Acetobacter* sp.

### 3.9 Molecular docking

Molecular docking helps visualize how small molecule ligands interact with receptors, which may further validate the previous experimental results (Charak et al., 2012). As a highly automated tool, molecular docking can not only predict ligand-receptor binding patterns and the most efficient sites for binding, but also calculate the binding energy of ligand-receptor interactions using more detailed molecular mechanics (Sarwar et al., 2015). To validate the binding mode of proanthocyanidins to DNA, as determined in previous experiments, molecular docking analysis was conducted following the experimental protocol described earlier. Generally, a docking result was considered reliable when the binding free energy was below −1.2 kcal/mol (Khajeh et al., 2018). The obtained results are presented in Figures 6C,D. The proanthocyanidins were shown to have optimal fit within the minor groove of DNA, exhibiting a minimal binding energy of −6.88 kcal/mol, thereby indicating a relatively high binding potential for the proanthocyanidin-DNA complex. The binding site was identified in the region rich in Adenine and Thymine, with interactions facilitated by multiple hydrogen bonds. In this conformation, proanthocyanidins primarily interacted with Guanine (DT-8) and Thymine (DG-10) on one of the chains of the DNA double helix via one and three hydrogen bonds, respectively. At the same time, it interacted with Thymine (DT-19, DT-20) on the other chain by three hydrogen bonds. Hence, the complementarity of previous spectroscopic experiments and molecular docking results further confirmed that proanthocyanidins and DNA bound to each other through a minor groove pattern. Regardless of the mechanism by which proanthocyanidins may enter the *Acetobacter* sp. cells, it was shown that there was extensive interaction between proanthocyanidins and DNA dodecamerer, and they interacted through minor grooves. Further, this combination may potentially block DNA replication pathways, thereby inhibiting the proliferation of *Acetobacter* sp.

## 4 Conclusion

In this study, the inhibition of proanthocyanidins on the growth of *Acetobacter* sp. was investigated, and the antibacterial mechanism of proanthocyanidins against *Acetobacter* sp. was fully elucidated. The results showed that a certain concentration of proanthocyanidins could inhibit the activity of *Acetobacter* sp. and delay the arrival time of *Acetobacter* sp. to the stationary phase, with a MIC of 2.5 mg/mL. Furthermore, proanthocyanidins disrupted cell morphology and cell membrane integrity in *Acetobacter* sp., leading to the leakage of nucleotides and proteins. Moreover, the antibacterial effects of proanthocyanidins against *Acetobacter* sp. may be partly achieved by causing oxidative damage to the cell membrane, affecting the structure of membrane proteins and altering the composition ratio of membrane fatty acids. After crossing the cell membrane barrier, proanthocyanidins could decrease the activity of ADH/ALDH, thereby inhibiting the acid-producing capacity of *Acetobacter* sp.. In addition, it interacted with DNA through the groove binding mode and potentially block DNA replication pathways, inhibiting cell function and ultimately leading to cell death. The results indicated that proanthocyanidins represent an attractive and promising bacteriostatic agent with significant inhibitory effects against *Acetobacter* sp.

## Data Availability

The original contributions presented in the study are included in the article/supplementary material, further inquiries can be directed to the corresponding author.

## References

[B1] Alvarez-Ordonez A. Fernandez A. Lopez M. Arenas R. Bernardo A. (2008). Modifications in membrane fatty acid composition of *Salmonella typhimurium* in response to growth conditions and their effect on heat resistance. *Int. J. Food Microbiol.* 123 212–219. 10.1016/j.ijfoodmicro.2008.01.015 18313782

[B2] Bajpai V. Sharma A. Baek K. (2013). Antibacterial mode of action of *Cudrania tricuspidata* fruit essential oil, affecting membrane permeability and surface characteristics of food-borne pathogens. *Food Control* 32 582–590. 10.1016/j.foodcont.2013.01.032

[B3] Bisha B. Weinsetel N. Brehm-Stecher B. Mendonca A. (2010). Antilisterial effects of gravinol-s grape seed extract at low levels in aqueous media and its potential application as a produce wash. *J. Food Prot.* 73 266–273. 10.4315/0362-028X-73.2.266 20132671

[B4] Charak S. Shandilya M. Tyagi G. Mehrotra R. (2012). Spectroscopic and molecular docking studies on chlorambucil interaction with DNA. *Int. J. Biol. Macromol.* 51 406–411. 10.1016/j.ijbiomac.2012.06.012 22710244

[B5] Coulon J. Seabrook A. (2020). Fermentation: Low SO2 winemaking-microbial control post-fermentation. *Wine Vitic. J.* 35 22–25. 10.3316/informit.943635267779948

[B6] Di Pasqua R. Hoskins N. Betts G. Mauriello G. (2006). Changes in membrane fatty acids composition of microbial cells induced by addiction of thymol, carvacrol, limonene, cinnamaldehyde, and eugenol in the growing media. *J. Agric. Food Chem.* 54 2745–2749. 10.1021/jf052722l 16569070

[B7] Ebrahimipour S. Sheikhshoaie I. Mohamadi M. Suarez S. Baggio R. Khaleghi M. (2015). Synthesis, characterization, X-ray crystal structure, DFT calculation, DNA binding, and antimicrobial assays of two new mixed-ligand copper (II) complexes. *Spectrochim. Acta A Mol. Biomol. Spectrosc.* 142 410–422. 10.1016/j.saa.2015.01.088 25725448

[B8] Elshikh M. Ahmed S. Funston S. Dunlop P. McGaw M. Marchant R. (2016). Resazurin-based 96-well plate microdilution method for the determination of minimum inhibitory concentration of biosurfactants. *Biotechnol. Lett.* 38 1015–1019. 10.1007/s10529-016-2079-2 26969604 PMC4853446

[B9] Feng Y. Yang T. Zhang Y. Zhang A. Gai L. Niu D. (2022). Potential applications of pulsed electric field in the fermented wine industry. *Front. Nutr.* 9:1048632. 10.3389/fnut.2022.1048632 36407532 PMC9668251

[B10] Guan Y. Zhou W. Yao X. Zhao M. Li Y. (2006). Determination of nucleic acids based on the fluorescence quenching of Hoechst 33258 at pH 4.5. *Anal. Chim. Acta* 570 21–28. 10.1016/j.aca.2006.03.106

[B11] Han Y. Sun Z. Chen W. (2020). Antimicrobial susceptibility and antibacterial mechanism of limonene against *Listeria monocytogenes*. *Molecules* 25:33. 10.3390/molecules25010033 31861877 PMC6982812

[B12] Hu Y. Xie M. Wu X. (2019). Interaction studies of sodium cyclamate with DNA revealed by spectroscopy methods. *Spectrochim. Acta A Mol. Biomol. Spectrosc.* 220:117085. 10.1016/j.saa.2019.04.077 31146213

[B13] Jekabsone A. Sile I. Cochis A. Makrecka-Kuka M. Laucaityte G. Makarova E. (2019). Investigation of antibacterial and antiinflammatory activities of proanthocyanidins from *Pelargonium sidoides* DC root extract. *Nutrients* 11:2829. 10.3390/nu11112829 31752295 PMC6893413

[B14] Johnson L. Hug L. A. (2019). Distribution of reactive oxygen species defense mechanisms across domain bacteria. *Free Radic. Biol. Med.* 140 93–102. 10.1016/j.freeradbiomed.2019.03.032 30930298

[B15] Khajeh M. Dehghan G. Dastmalchi S. Shaghaghi M. Iranshahi M. (2018). Spectroscopic profiling and computational study of the binding of tschimgine: A natural monoterpene derivative, with calf thymus DNA, spectrochim. *Acta A Mol. Biomol. Spectrosc.* 192 384–392. 10.1016/j.saa.2017.11.042 29195192

[B16] Li J. Wang J. Fan J. Huang G. Yan L. (2020). Binding characteristics of aflatoxin B-1 with free DNA in vitro. *Spectrochim. Acta A Mol. Biomol. Spectrosc.* 230:18054. 10.1016/j.saa.2020.118054 32006841

[B17] Lisanti M. Blaiotta G. Nioi C. Moio L. (2019). Alternative methods to SO2 for microbiological stabilization of wine. *Compr. Rev. Food Sci. Food Saf.* 18 455–479. 10.1111/1541-4337.12422 33336947

[B18] Liu B. Zhang J. Wang X. Zhang L. Liu Y. Niu H. (2015). Synthesis, DNA interaction and antimicrobial activities of three rimantadine analogues. *J. Lumin.* 159 128–133. 10.1016/j.jlumin.2014.11.008

[B19] Liu X. Cai J. Chen H. Zhong Q. Hou Y. Chen W. (2020). Antibacterial activity and mechanism of linalool against *Pseudomonas aeruginosa*. *Microb. Pathog.* 141:103980. 10.1016/j.micpath.2020.103980 31962183

[B20] Liu X. Jiang X. Zang L. (2011). In vitro study on the effect of grape seed proanthocycandin extract (GSPE) on *Actinomyces viscosus*. *J. Pract. Stomatol.* 27 761–764. 10.3969/j.issn.1001-3733.2011.06.06

[B21] Luz I. D. S. de Melo A. N. F. Bezerra T. K. A. Madruga M. S. Magnani M. de Souza E. L. (2014). Sublethal amounts of *Origanum vulgare* L. essential oil and carvacrol cause injury and changes in membrane fatty acid of *Salmonella typhimurium* cultivated in a meat broth. *Foodborne Pathog. Dis.* 11 357–361. 10.1089/fpd.2013.1695 24588810

[B22] Lv F. Liang H. Yuan Q. Li C. (2011). In vitro antimicrobial effects and mechanism ofaction of selected plant essential oil combinations against four food-related microorganisms. *Food Res. Int.* 44 3057–3064. 10.1016/j.foodres.2011.07.030

[B23] Matuschek E. Ahman J. Webster C. Kahlmeter G. (2018). Antimicrobial susceptibility testing of colistin-evaluation of seven commercial MIC products against standard broth microdilution for *Escherichia coli*, *Klebsiella pneumoniae*, *Pseudomonas aeruginosa*, and *Acinetobacter* spp. *Clin. Microbiol. Infect.* 24 865–870. 10.3390/molecules25010033 29221995

[B24] Moghimi R. Ghaderi L. Rafati H. Aliahmadi A. McClements D. (2016). Superior antibacterial activity of nanoemulsion of *Thymus daenensis* essential oil against *E. coli*. *Food Chem.* 194 410–415. 10.1016/j.foodchem.2015.07.139 26471573

[B25] Mukherjee A. Singh B. (2017). Binding interaction of pharmaceutical drug captopril with calf thymus DNA: A multispectroscopic and molecular docking study. *J. Lumin.* 190 319–327. 10.1016/j.jlumin.2017.05.068

[B26] Niu D. Ren E. Li J. Zeng X. Li S. (2021). Effects of pulsed electric field-assisted treatment on the extraction, antioxidant activity and structure of naringin. *Sep. Purif. Technol.* 265:118480. 10.1016/j.seppur.2021.118480

[B27] Niu D. Wang L. Zeng X. Wen Q. Brennan C. Tang Z. (2019a). Effect of ethanol adaption on the inactivation of *Acetobacter* sp. by pulsed electric fields. *Innov. Food Sci. Emerg. Technol.* 52 25–33. 10.1016/j.ifset.2018.11.009

[B28] Niu D. Wang Q. Ren E. Zeng X. Wang L. He T. (2019b). Multi-target antibacterial mechanism of eugenol and its combined inactivation with pulsed electric fields in a hurdle strategy on *Escherichia coli*. *Food Control* 106:106742. 10.1016/j.foodcont.2019.106742

[B29] Niu D. Zeng X. Ren E. Xu F. Li J. Wang M. (2020). Review of the application of pulsed electric fields (PEF) technology for food processing in China. *Food Res. Int.* 137:109715. 10.1016/j.foodres.2020.109715 33233287

[B30] Patrignani F. Lucci L. Belletti N. Gardini F. Guerzoni A. Lanciotti R. (2008). Effects of sub-lethal concentrations of hexanal and 2-(E)-hexenal on membrane fatty acid composition and volatile compounds of *Listeria monocytogenes*, *Staphylococcus aureus*, *Salmonella enteritidis* and *Escherichia coli*. *Int. J. Food Microbio.* 123 1–8. 10.1016/j.ijfoodmicro.2007.09.009 18055050

[B31] Pei J. Jiang L. Dai H. Chen P. (2016). Application of nisin-the well-known lactic acid bacteria bacteriocin-against spoilage bacteria in tangerine wine. *Czech J. Food Sci.* 34 488–494. 10.17221/545/2015-CJFS

[B32] Rapacka-Zdonczyk A. Wozniak A. Michalska K. Pieranski M. Ogonowska P. Grinholc M. (2021). Factors determining the susceptibility of bacteria to antibacterial photodynamic inactivation. *Front. Med.* 8:642609. 10.3389/fmed.2021.642609 34055830 PMC8149737

[B33] Rauf A. Imran M. Abu-Izneid T. Iahfisham-Ul-Haq Patel S. Pan X. Naz S. (2019). Proanthocyanidins: A comprehensive review. *Biomed. Pharmacother.* 116:108999. 10.1016/j.biopha.2019.108999 31146109

[B34] Rehman S. Sarwar T. Ishqi H. Husain M. Hasan Z. Tabish M. (2015). Deciphering the interactions between chlorambucil and calf thymus DNA: A multi-spectroscopic and molecular docking study. *Arch. Biochem. Biophys.* 566 7–14. 10.1016/j.abb.2014.12.013 25528167

[B35] Sarwar T. Rehman S. Husain M. Ishqi H. Tabish M. (2015). Interaction of coumarin with calf thymus DNA: Deciphering the mode of binding by in vitro studies. *Int. J. Biol. Macromol.* 73 9–16. 10.1016/j.ijbiomac.2014.10.017 25453293

[B36] Sasser M. (2006). *Bacterial identification by gas chromatographic analysis of fatty acids methyl esters (GC-FAME).* Newark, NY: MIDI-Inc.

[B37] Tagousop C. Tamokou J. Ekom S. Ngnokam D. Voutquenne-Nazabadioko L. (2018). Antimicrobial activities of flavonoid glycosides from *Graptophyllum grandulosum* and their mechanism of antibacterial action. *BMC Complement. Altern. Med.* 18:252. 10.1016/j.jlumin.2014.11.008 30219066 PMC6139119

[B38] Tedesco F. Siesto G. Pietrafesa R. Romano P. Salvia R. Scieuzo C. (2022). Chemical methods for microbiological control of winemaking: An overview of current and future applications. *Beverages* 8:58. 10.3390/beverages8030058

[B39] Ulrey R. Barksdale S. Zhou W. van Hoek M. (2014). Cranberry proanthocyanidins have anti-biofilm properties against *Pseudomonas aeruginosa*. *BMC Complement. Altern. Med.* 14:499. 10.1186/1472-6882-14-499 25511463 PMC4320558

[B40] Valera M. Sainz F. Mas A. Torija M. (2017). Effect of chitosan and SO2 on viability of *Acetobacter* strains in wine. *Int. J. Food Microbiol.* 246 1–4. 10.1016/j.ijfoodmicro.2017.01.022 28187326

[B41] Vavrinik A. Stuskova K. Baron M. Sochor J. (2022). Effect of sulphur dioxide and ethanol on acetic acid bacteria occurring in wine technology. *J. Food Nutr. Res.* 61 209–217.

[B42] Wang J. Wang H. (2017). Antibacterial activities of grape seed procyanidins against *Streptococus mutans* in vitro. *J. Nanjing Univ. Tradit. Chin. Med.* 33 173–176. 10.14148/j.issn.1672-0482.2017.0173

[B43] Wang L. Wang M. Zeng X. Xu X. Brennan C. (2017a). Membrane and genomic DNA dual-targeting of citrus flavonoid naringenin against: *Staphylococcus aureus*. *Integr. Biol.* 9 820–829. 10.1039/c7ib00095b 28862705

[B44] Wang L. Zhang Z. Zeng X. Gong D. Wang M. (2017b). Combination of microbiological, spectroscopic and molecular docking techniques to study the antibacterial mechanism of thymol against *Staphylococcus aureus*: Membrane damage and genomic DNA binding. *Anal. Bioanal. Chem.* 409 1615–1625. 10.1007/s00216-017-0264-3 27900434

[B45] Wang Y. Malkmes M. Jiang C. Wang P. Zhu L. Zhang H. (2021). Antibacterial mechanism and transcriptome analysis of ultra-small gold nanoclusters as an alternative of harmful antibiotics against Gram-negative bacteria. *J. Hazard. Mater.* 416:126236. 10.1016/j.foodcont.2019.106742 34492988

[B46] Wu X. Yang M. Kim J. Wang R. Kim G. Ha J. (2022). Reactivity differences enable ROS for selective ablation of bacteria. *Angew. Chem. Int. Ed.* 134:e202200808. 10.1002/anie.202200808 35174598

[B47] Xu Y. Guan X. Wang S. (2024). Synergistic bactericidal mechanisms of RF energy simultaneously combined with cinnamon essential oil or epsilon-polylysine against *Salmonella* revealed at cellular and metabolic levels. *Int. J. Food Microbiol.* 408:110447. 10.1016/j.ijfoodmicro.2023.110447 37907022

[B48] Ye X. Li X. Yuan L. Ge L. Zhang B. Zhou S. (2007). Interaction of houttuyfonate homologues with the cell membrane of gram-positive and gram-negative bacteria. *Colloids Surf. A Physicochem. Eng. Asp.* 301 412–418. 10.1016/j.colsurfa.2007.01.012

[B49] Yin L. Chen J. Wang K. Geng Y. Lai W. Huang X. (2020). Study the antibacterial mechanism of cinnamaldehyde against drug-resistant *Aeromonas hydrophila* in vitro. *Microb. Pathog.* 145:104208. 10.1016/j.micpath.2020.104208 32325237

[B50] Zhou D. Wang D. Yang L. Liu Z. Zhang Y. W. (2016). A modified and improved assay based on microbial test system (MTS) to evaluate antioxidant activity. *Food Anal. Methods* 9 895–904. 10.1007/s12161-015-0266-8

[B51] Zhu C. Guan F. Wang C. Jin L. (2014). The protective effects of *Rhodiola crenulata* extracts on *Drosophila melanogaster* gut immunity induced by bacteria and SDS toxicity. *Phytother. Res.* 28 1861–1866. 10.1002/ptr.5215 25146450

[B52] Zhu B. Xia X. Xia N. Zhang S. Guo X. (2014). Modification of fatty acids in membranes of bacteria: Implication for an adaptive mechanism to the toxicity of carbon nanotubes. *Environ. Sci. Technol.* 48 4086–4095. 10.1021/es404359v 24579825

